# Supplementary material for: Bayesian refinement of protein structures and ensembles against SAXS data using molecular dynamics
Source: PLoS Comput Biol. 2017 Oct 18;13(10):e1005800. doi: 10.1371/journal.pcbi.1005800 (PMC5662244; doi:10.1371/journal.pcbi.1005800)
Supplement: S1 Archive — (BZ2) [file pcbi.1005800.s011.tar.bz2 › gromacs_4.62_bayesian_saxs_refi/share/html/gmxfaq.html]

GROMACS FAQ


| GROMACS FAQ Main Table of Contents |

**VERSION 4.5  
Thu 26 Aug 2010**

---

If you don't find the solution to your problem here, you could have a look in
the online archives of our 
mailing lists, or subscribe yourself!

There is also a special Developer FAQ
at www.gromacs.org with more advanced and/or technical topics (e.g. automake/autoconf) available under
Developer info that you could use, and when all else fails it's
time to post your question to the mailing lists!

### Download & Installation

- How can I get GROMACS and how much does it cost?- Is my system supported?- Can't you provide binaries for my system?- How do I compile and install the GROMACS package?- How do I select the compiler and/or flags to use?- The configuration script complains about FFTW - how
            do I install it?- I HAVE installed FFTW, but the configuration script
              still says it can't find it!- How do I compile GROMACS for parallel runs?- When I enable MPI support for parallel runs, GROMACS
                  looks for a special MPI wrapper script like 'mpicc', but we don't use that; is it possible to
                  add the MPI library manually with -lmpi ?- How do I turn off Motif?- Everything compiles fine, but when I try to run a program
                      it complains about not finding libXXXX.so.- I get an error from the configure script on Mac OS X!- It still won't compile and I haven't got a clue what the problem might be...- How fast is GROMACS compared to other programs?- Is there any way I can make GROMACS run faster?- What hardware do you recommend?- How large systems can I simulate with GROMACS?- Why is the front page graphics in the PDF manual strange?

  

### System preparation

- OK, I've downloaded a PDB file with a structure I'd like
  to simulate. What should I do?- My protein has multiple subunits. Is that a problem?- How do I convert my structure from a .gro, .tpr, or trajectory
      file to a .pdb file?- The pdb2gmx program is complaining about long bonds and/or
        missing atoms. What should I do?- grompp doesn't find the C preprocessor /lib/cpp on OS X!

  

### Simulation

- What does "1-4 (#,#) interaction not within cut-off" mean?- What does "Fatal error: Routine gmx\_tx called in libnet.c" mean?- My simulation seems to be running, but shouldn't there be any output?- Why do I get very strange temperatures in my simulation?- Is there any smart way to continue a run that crashed?- When my trajectory files reach 2GB I get strange error messages,
            or they just disappear. Why?

  

### Analysis

- How do I analyze a PDB file with multiple entries?- Can I fit two structures which do not have the
    same number/sequence of atoms?- I get tired of having to select the same index group
      over and over again. Is there a better way to do it?- How do I perform an analysis that GROMACS doesn't have a program
        for?

  

### Other problems

- My problem isn't mentioned above, and/or none of the solutions seem to work?

  
  
  
  


---

- **How can I get GROMACS and how much does it cost?**  
    
  You can download it immediately from this website, and it won't cost
  you a penny! GROMACS is free software, licensed under the GNU General
  Public License. The details are available in the
  license text, but
  in short you can modify and redistribute the code as long as your
  version is licensed under the GPL too.   
    
  - **Is my system supported?**  
      
    GROMACS is a recursive acronym for "GROMACS Runs On Most Of All Computer Systems" :-)  
    Since we use GNU automatic configuration scripts you should in principle
    be able to compile GROMACS on any UNIX dialect, probably including Mac OS X.
    Contact us if you have any problems. At least Solaris, IRIX, Linux (both x86 and alpha),
    Tru64/Digital Unix, and AIX should be virtually problem-free. An ANSI C compiler
    is sufficient to compile GROMACS, but we definitely recommend a good Fortran 77
    compiler too (performance-critical routines are available in fortran versions). You
    won't need Fortran on Linux/x86 where we provide even faster assembly loops!  
      
    - **Can't you provide binaries for my system?**  
        
      The problem is that we want the highest possible performance, and to achieve this
      it is necessary to adapt compiler flags to your processor type. We also use special
      mathematic libraries from several hardware manufacturers, and different versions
      of MPI for parallel runs. This means we would have to provide about 10 different
      sets of binaries for each processor on each operation system, and keep them updated
      for each new release. Sorry, but that's simply not possible.  
      However, for Linux running on x86 computers it doesn't matter which compiler
      flags we use since it doesn't affect the assembly loops, and we can thus distribute
      RPM packages of GROMACS. If you want to run in parallel you probably have to install our
      versions of the LAM MPI packages to get the correct version, or compile GROMACS yourself.
        
        
      - **How do I compile and install the GROMACS package?**  
          
        That's easy - we provide step-by-step instructions for you here.  
        If you're impatient you could just unpack the distribution and try  
          

        |  |  |  |
        | --- | --- | --- |
        |  | ./configure  make  make install  \| |

          
        The configure script will complain if it doesn't find FFTW, but you will be told what to do.  
          
        This setup is new from version 3.0, so there might be some bugs we've missed, though. Don't
        hesitate to post questions to the 
        **How do I select the compiler and/or flags to use?**  
          
        Select the compiler by setting the CC and/or F77 environment variables before running the
        GROMACS configure script (MPICC for the MPI C compiler). You can also set the corresponding compiler flags with CFLAGS and
        FFLAGS, and the linker flags with LDFLAGS. If you want to add a library at the link stage
        you can add the -llib flags to the LIBS variable, and include directories can be
        added in the CPPFLAGS variable.  
          
        - **The configuration script complains about FFTW - how
          do I install it?**  
            
          FFTW uses the same type of automatic configuration scripts as
          GROMACS, so it's easy to configure and compile. The default setup
          places libraries under /usr/local, but you can change it with
          --prefix. One important difference is that GROMACS normally
          is compiled in single precision, while FFTW defaults to double precision.
          Configure and install FFTW with the command:  
            

          |  |  |  |
          | --- | --- | --- |
          |  | ./configure --enable-float  make  make install  \| |

            
          If you want to compile GROMACS with parallel MPI support you should
          also add --enable-mpi to the FFTW configuration. Once the installation
          is ready we recommend that you also install a double-precision version
          of FFTW (nice to have) with:  
            

          |  |  |  |
          | --- | --- | --- |
          |  | make distclean  ./configure  make  make install  \| |

            
          That's it. Consult www.fftw.org if you have any problems.  
            
          - **I HAVE installed FFTW, but the configuration script
            still says it can't find it!**  
              
            OK. The problem is most probably that your compiler doesn't look for the
            header files and/or libraries in the place where you installed them. It's easy to fix; you
            can just tell the GROMACS configure script to add those directories to the search paths.
            Specify the header file directory (e.g. /home/erik/fftw/include) as  
              

            |  |  |  |
            | --- | --- | --- |
            |  | CPPFLAGS="-I/home/erik/fftw/include"  \| |

              
            and the location of the libraries (e.g. /home/erik/fftw/lib) in  
              

            |  |  |  |
            | --- | --- | --- |
            |  | LDFLAGS="-L/home/erik/fftw/lib"  \| |

              
            and then run the GROMACS configuration script. Note that some compilers don't search
            /usr/local by default, so you might have to specify these paths even if you installed
            FFTW in the default place.  
              
            - **How do I compile GROMACS for parallel runs?**  
                
              On most systems you only have to add the option --enable-mpi to the configure
              script, and then compile GROMACS the normal way. For this to work you need to
              have MPI communication libraries install, and some kind of wrapper script like
              mpicc or mpcc to use when compiling MPI programs. MPI should always be present
              on supercomputers, and if you are running on workstations we recommend LAM MPI,
              www.lam-mpi.org. On Linux it's even simpler -
              just install the RPM packages we provide!  
                
              On most supercomputers you can only run MPI programs on dedicated nodes, so
              in this case you probably want all the analysis programs compiled without MPI first.
              Since you normally only need the mdrun program with MPI support you can type
              "make mdrun; make install-mdrun" instead, but remember to type "make distclean"
              if you have previously compiled GROMACS without MPI. It is also possible to put a suffix
              on the MPI-version programs, or just start MPI runs when an environment variable
              is set. Check the options to the configure script with "./configure --help".
                
                
              - **When I enable MPI support for parallel runs, GROMACS
                looks for a special MPI wrapper script like 'mpicc', but we don't use that; is it possible to
                add the MPI library manually with -lmpi ?**  
                  
                Sure - no problem, but it might not be entirely obvious if you are new to autoconf scripts.
                Here's how to do it:  
                  
                To use the MPI library we need the header files (mpi.h) with definitions, and the MPI libraries
                with the actual code (e.g. libmpi.a). If your system uses some special hardware it might
                also be necessary to link with more libraries - ask your system administrator if you have
                any problems. Start by location these headers and libraries on your system, and then add
                them to your environment variables before running the configure script:
                  
                  

                |  |  |  |
                | --- | --- | --- |
                |  | setenv CPPFLAGS "-I/path/to/your/mpi/include"  setenv LDFLAGS "-L/path/to/your/mpi/lib"  setenv LIBS "-lmpi"  setenv MPICC "cc"  \| |

                  
                (This is valid for tcsh, for bash you should use export instead.) Note that these commands overwrite
                any previous assignments, so you must add
                all parts you want (you can use $VARIABLE to add the previous value of an environment variable).
                  
                Now you should be able to run ./configure --enable-mpi !
                  
                  
                - **How do I turn off Motif?**  
                    
                  Just use the flag --without-motif-libraries (or headers).
                  If the configure script doesn't find both libraries and headers it will disable motif. This is useful when you have motif on the machine where you compile, but not on all machines you run on.  
                    
                  - **Everything compiles fine, but when I try to run a program
                    it complains about not finding libXXXX.so.**  
                      
                    GROMACS and/or the FFTW package can be compiled with shared libraries. In fact,
                    it's the default setup in the Linux RPMs. This means we save space by not linking all the
                    routines into each binary, but call the shared library at runtime. Of course, this requires that you can find the library at runtime. For the GROMACS distribution programs we hardcode the location of the GROMACS and FFTW libraries, but if you compile your own programs or move your libraries you must tell the system where to find them!
                    Fortunately, this is quite easy to do. On Linux you can do it permanently for
                    all users if you are root, by adding the search path to the file /etc/ld.so.conf. Alternatively, you can add it to the LD\_LIBRARY\_PATH environment variable:
                      
                      

                    |  |  |  |
                    | --- | --- | --- |
                    |  | setenv LD\_LIBRARY\_PATH "$LD\_LIBRARY\_PATH:/opt/lib"  \| |

                      
                    (This is valid for tcsh, for bash you should use export instead.) Ask your
                    local sysadm how to add it to your login file so it's done automatically each
                    time you log on.
                      
                      
                    - **I get an error from the configure script on Mac OS X!**  
                        
                      This is because OS X uses Z shell for /bin/sh. This will hopefully be
                      fixed in a future release of automake (it is not caused by Gromacs), but in the
                      meantime you can install bash (if you don't already have it) and
                      use the command '/bin/bash ./configure' (Your bash location might be different from /bin/bash).
                        
                        
                      - **It still won't compile and I haven't got a clue what the problem might be...**  
                          
                        Oops. Sorry, but those things happen :-) It's usually quite easy to fix. One possible
                        source of errors is the shared libraries we use to save space. You can try to disable
                        them with the --disable-shared flag to the configure script. You can also ask questions on the mailing
                        lists or contact us. BUT: Unless you attach copies of your configuration/make output and/or
                        log files we can only guess what your problem might be!  
                          
                        - **How fast is GROMACS compared to other programs?**  
                            
                          GROMACS is fast, VERY fast. In fact, on all benchmarks we've tested it's
                          3-10 times faster than any other program we've tried, many of which are
                          commercial. On x86 hardware GROMACS really excels due to the assembly loops.
                          Of course, speed isn't everything. There are cases where
                          we don't support a certain algorithm that program X supports, and
                          vice versa. For instance, our assembly loops are only available in single precision.
                          In any case - show us a benchmark were some other program
                          is faster and we'll be happy to implement that algorithm.   
                            
                          - **Is there any way I can make GROMACS run faster?**  
                              
                            That depends on your setup. If you are using x86 processors you should definitely
                            make sure that you compile GROMACS with assemblt loops, and that your OS
                            supports SSE instructions if you are using Pentium III/IV processors. If you
                            compiled GROMACS with assembly loops there will be a line in the logfile telling
                            you which loops we are using.  
                            On alpha hardware you might want to play around with enabling/disabling the
                            software invsqrt, and the inner loop vectorization. Modern alpha chips have a
                            fairly fast hardware sqrt, but they also seem to benefit even more from vectorizing the
                            innerloops and using the vectorized invsqrt provided in GROMACS.  
                            If you are using IBM hardware you should locate or download the MASS libraries
                            (mathematical accelerated subsystem). If you provide the location of this library
                            in the LDFLAGS environment variable GROMACS will automatically use fast vectorized
                            inner loops on IBM.  
                            On any system apart from Linux/x86 (where we use assembly innerloops) you should also
                            try to use a fortran compiler for better performance, and if you run Linux/alpha
                            you should use the Compaq compilers instead of gcc.  
                              
                            You should always use single precision; there are very few cases where you
                            actually need double precision, and it's slower.  
                              
                            Investigate the options -dummy and -heavyh to
                            pdb2gmx
                            which control the constraining of hydrogen atoms and the mass of
                            unconstrained hydrogen atoms. This eliminates the highest freqency
                            motions in your system, enabling you to increase the timestep without
                            loss of accuracy to about 4 fs, or even up to 7 fs with negligible
                            loss of accuracy! (*J. Comput. Chem.* **20**,786).  
                              
                            If your system has relatively slow disk-IO, and/or you write
                            frames and energies out very often, and/or you have a very large
                            system the performance might be limited by disk access. In that case,
                            you might consider writing fewer frames to your trajectories
                            (.xtc and especially .trr or .trj) and
                            energy file (.ene or .edr).  
                              
                            - **What hardware do you recommend?**  
                                
                              If cost is an issue, you can't beat dual Pentium boxes due to the assembly loops!
                              Dual AMD machines should also be a nice option soon. Note that the Pentium 4 processors
                              achieve a high clock frequency by using a longer pipeline, so a Pentium 4 at 1.7 GHz is
                              about the same speed as a Pentium III at 1.2 GHz. Don't be fooled by the high clock!  
                                
                              - **How large systems can I simulate with GROMACS?**  
                                  
                                It's only limited by your memory, and GROMACS is quite modest in its
                                memory requirements. As an indictaion: a system of 12000 atoms takes
                                about 10Mb of memory, and 6000 atoms about 5.5Mb (on a SGI O2),
                                which comes down to just over 900 bytes memory use per atom in
                                your system (your mileage will vary). Due to the fact that we initially
                                developed GROMACS to run on our home-built parallel machine, with
                                only 8Mb of memory per processor, the code is quite well optimized for
                                memory use. To get an indication of scaling of GROMACS performance as
                                a function of system size, have a look at the
                                scaling benchmark page.  
                                  
                                Note that the .gro format only support atoms numbers up to 99999, so
                                it will loop once it reaches 100000 atoms. This is no problem in GROMACS
                                since we don't use the atom number from the .gro file.  
                                  
                                - **Why is the front page graphics in the PDF manual strange?**  
                                    
                                  This is a known problem with some versions of Acrobat reader on Linux. There
                                  is nothing we can do about it, but in the cases we've tested the graphics
                                  still prints fine on paper.  
                                    
                                  - **OK, I've downloaded a PDB file with a structure I'd like
                                    to simulate. What should I do?**  
                                      
                                    Look at the flowchart for a quick overview.
                                    Start where it says "eiwit.pdb" (this is somewhere at the top).
                                    More detailed info can be found in the
                                    Getting Started
                                    section, you can probably start where it says "Ribonuclease S-Peptide".
                                      
                                      
                                    - **My protein has multiple subunits. Is that a problem?**  
                                        
                                      pdb2gmx can automatically process multimeric proteins,
                                      but won't be able to make inter-subunit cystine bridges. A word of warning, though:
                                      the units will only be recognized as different chains if they
                                      have different chain identifiers!  
                                        
                                      - **How do I convert my structure from a .gro, .tpr, or trajectory
                                        file to a .pdb file?**  
                                          
                                        Any generic structure file,
                                        for instance .gro, .pdb or .tpr, can be
                                        converted to .pdb with
                                        editconf. You can view a
                                        .pdb file with several programs, for instance rasmol. Two generic
                                        structure files can be fitted with
                                        g\_confrms, the two
                                        superimposed structures can be written to a .pdb file. Any
                                        generic trajectory format can be
                                        converted with trjconv.
                                        You can dump one frame with trjconv -dump, or write a
                                        .pdb with multiple frames using trjconv -op -app.
                                        If multiple structures in a .pdb are separated by
                                        ENDMDL keywords, you should use rasmol -nmrpdb to
                                        view them.  
                                          
                                        - **The pdb2gmx program is complaining about long bonds and/or
                                          missing atoms. What should I do?**  
                                            
                                          There are probably atoms missing earlier in the
                                          .pdb file
                                          which makes pdb2gmx
                                          go crazy. Check the screen output of pdb2gmx, as it
                                          will tell you which one is missing. Then add the atoms in your pdb file,
                                          energy minimization will put them in the right place,
                                          or fix the side chain with e.g. the
                                          WhatIF program.  
                                            
                                          - **grompp doesn't find the C preprocessor /lib/cpp on OS X!**  
                                              
                                            Yep, that's right. OS X is a real Unix system, but Apple have been moving
                                            some stuff around. Look for it in /usr/bin or possible a place like
                                            /usr/libexec/gcc/darwin/ppc/2.95.2/cpp. Since a lot of programs assume
                                            cpp to be present in /lib it is probably smart to make a link, but you
                                            can also specify the location with the cpp keyword in your mdp files.
                                              
                                              
                                            - **What does "1-4 (#,#) interaction not within cut-off" mean?**  
                                                
                                              Some of your atoms have moved so two atoms separated by three bonds are
                                              separated by more than the cutoff distance. This is BAD.
                                              Most important: **do not increase your cut-off!** This error
                                              actually indicates that the atoms have very large velocities,
                                              which usually means that (part of) your molecule(s) is (are)
                                              exploding. If you are using LINCS for constraints, you probably also
                                              already got a number of LINCS warnings. When using SHAKE this will
                                              give rise to a SHAKE error, which halts your simulation before the
                                              "1-4 not within cutoff" error can appear.   
                                                
                                              There can be a number of reasons for the large velocities in
                                              your system. If it happens at the
                                              beginning of the simulation, your system might be not equilibrated
                                              well enough (e.g. it contains some bad contacts). Try a(nother) round
                                              of energy minimization to fix this. Otherwise you might have a very
                                              high temperature, and/or a too large
                                              timestep. Experiment with these parameters till the error stops
                                              occurring. If this doesn't help, check your topology!  
                                                
                                              - **What does "Fatal error: Routine gmx\_tx called in libnet.c" mean?**  
                                                  
                                                You probably made a parallel mdrun without typing make distclean. The error
                                                messages tells you that the GROMACS library you are using doesn't support parallel runs.  
                                                  
                                                - **My simulation seems to be running, but shouldn't there be any output?**  
                                                    
                                                  - Your simulation might simply be (very) slow, and since output is
                                                    buffered, it can take quite some time for output to appear in the
                                                    respective files. If you are trying to fix some problems and you want
                                                    to get output as fast as possible, you can set the environment
                                                    variable LOG\_BUFS to 0 by using setenv LOG\_BUFS 0,
                                                    this disables output buffering. Use unsetenv LOG\_BUFS to turn
                                                    buffering on again.- Something might be going wrong in your simulation, causing
                                                      e.g. not-a-numbers (NAN) to be generated (these are
                                                      the result of e.g. division by zero). Subsequent calculations with
                                                      NAN's will generate floating point exceptions which slow
                                                      everything down by orders of magnitude. On a SGI system this will
                                                      usually result in a large percentage of CPU time being devoted to
                                                      'system' (check it with osview, or for a multi-processor
                                                      machine with top and osview).- You might have all
                                                        nst\* parameters (see
                                                        your .mdp file) set to 0,
                                                        this will suppress most output.- Your disk might be full. Eventually this will lead to
                                                          mdrun crashing, but since output is buffered, it might take a
                                                          while for mdrun to realize it can't write.- You are runnning an executable compiled with MPI support (e.g.
                                                            LAM) and did not start the LAM daemon
                                                            (lamboot). See LAM documentation.  
                                                    
                                                  - **Why do I get very strange temperatures in my simulation?**  
                                                      
                                                    You probably have very close contacts or a too large time
                                                    step. This causes inaccurate integration which will usually result in
                                                    a large positive temperature drift. Try some more energy minimization
                                                    to get rid of the close contacts, or if that still doesn't help, try a
                                                    short equilibration run with a small(er) time step.   
                                                      
                                                    - **Is there any smart way to continue a run that crashed?**  
                                                        
                                                      Yes, if the reason for the crash didn't have anything to doe with
                                                      the algorithms, i.e. it was due to a system crash, a full disk, or
                                                      a kill by the queuing system. Otherwise you'll have to use
                                                      grompp
                                                      and change the options.
                                                      To really continue a simulation as if nothing had happened, you will
                                                      need coordinates and velocities in full precision (i.e.
                                                      .trr format).
                                                      .xtc trajectories are in
                                                      reduced precision (only 3 decimal places after the decimal point) and
                                                      do not contain velocity information at all. Feed this trajectory and
                                                      your origional .tpr file to
                                                      tpbconv to obtain a new
                                                      .tpr file, *be sure*
                                                      to specify the one-but-last frame from your
                                                      .trr file, since the very
                                                      last frame is likely to be corrupted due to the crash. With the
                                                      .tpr file
                                                      tpbconv produces you can
                                                      restart your simulation.  
                                                        
                                                      After the continuation run is finished, you will have your simulation
                                                      split up in separate files, which you will probably want to combine.
                                                      This can be done as follows (the same command works for xtc-files):  
                                                        

                                                      |  |  |  |
                                                      | --- | --- | --- |
                                                      |  | trjcat -o whole.trr part1.trr part2.trr part3.trr  \| |

                                                        
                                                      The energy files can be concatenated in a similar manner:  
                                                        

                                                      |  |  |  |
                                                      | --- | --- | --- |
                                                      |  | eneconv -o whole.edr part1.edr part2.edr part3.edr  \| |

                                                        
                                                      Since tpbconv sets the time in the continuation runs the files are
                                                      automatically sorted and overlapping frames removed. If you have a mix of
                                                      runs continued with tpbconv and grompp you might have to set the times yourself
                                                      (see the manual pages for details).
                                                        
                                                        
                                                      It is of course possible to start a simulation from the coordinates in
                                                      your xtc file, but in that case new
                                                      velocities will have to be generated resulting in a 'kink' in the
                                                      simulation. To prevent this you should write coordinates and
                                                      velocities to a .trr file
                                                      during your simulations. Do this by setting
                                                      nstxout and
                                                      nstvout in your
                                                      .mdp file. You don't need
                                                      these frames very often (every 10 ps or so), but remember that when
                                                      mdrun crashes, everything
                                                      calculated after the last frame in the .trr file, will have to be
                                                      recalculated for a proper continuation.  
                                                        
                                                      - **When my trajectory files reach 2GB I get strange error messages,
                                                        or they just disappear. Why?**  
                                                          
                                                        This is a problem with the file system; when the system, or the C library,
                                                        or the compiler, or the NFS implementation (version 2) only uses 32 bits
                                                        for the file pointer you cannot use files larger than 2GB. On most modern
                                                        systems there are special compiler flags you can set to enable 64-bit file
                                                        pointers, but since the autoconf test for this doesn't work we have chosen
                                                        not to include any flags by default, since it can break other things. But
                                                        you can of course try anything if you add your own flags :-)
                                                          
                                                        In any case, it is probably a good idea to try to keep your files
                                                        smaller than 2GB. You never know if you later might need to use it over
                                                        NFS version 2 or on some supercomputer system that doesn't support large
                                                        files yet.  
                                                          
                                                        - **How do I analyze a PDB file with multiple entries?**  
                                                            
                                                          Assuming your
                                                          .pdb file is
                                                          called "eiwit.pdb", this is what you would do:  
                                                            

                                                          |  |  |  |
                                                          | --- | --- | --- |
                                                          |  | pdb2gmx -f eiwit.pdb -reth -ter -n  \| |

                                                            
                                                          -reth lets
                                                          pdb2gmx keep all
                                                          hydrogens which are present in your input file. It will also **not
                                                          add** any missing hydrogens, so your molecules should be
                                                          complete. -ter will cause pdb2gmx to ask for termini
                                                          types for which you must select 'none' for both C- and N-terminus.
                                                          -n tells pdb2gmx to generate a
                                                          .ndx file with the atoms
                                                          reordered to the GROMACS standard. pdb2gmx now generates a
                                                          topology file (topol.top)
                                                          which exactly corresponds with the molecule(s) in your input file.
                                                          It also writes a coordinate file
                                                          (conf.gro).  
                                                            

                                                          The next step is:
                                                            
                                                            

                                                          |  |  |  |
                                                          | --- | --- | --- |
                                                          |  | trjconv -f eiwit.pdb -o eiwit.xtc -n clean -timestep 1 -box 10 -center  \| |

                                                            
                                                          Yes, -f eiwit.pdb works because a .pdb is also a
                                                          trajectory format
                                                          in GROMACS. -ox sets output to
                                                          .xtc. -n clean
                                                          tells trjconv to
                                                          use the clean.ndx generated by
                                                          pdb2gmx, so the atom
                                                          ordering in the output (.xtc) file will be according to GROMACS
                                                          standards. -timestep 1 sets the timestep between output frames
                                                          to one, so the structures from the .pdb file get numbered
                                                          sequentially.
                                                          -ter causes TER markers in the .pdb
                                                          file to be seen as end-of-frame, default ENDMDL is used. If you
                                                          are not sure what is in your eiwit.pdb, TER is a good
                                                          guess, but you should check. If you have ENDMDL in stead of
                                                          TER, omit the -ter. -box 10 sets a default
                                                          box-size in the output .xtc trajectory (since no box is stored
                                                          in a .pdb file). The size is in nm and should be larger than
                                                          your molecule size. -center resets the geometrical center of
                                                          each of your structures to the center of the box (the one you specify
                                                          with -box). trjconv will generate a .xtc
                                                          trajectory file with all the coordinates from your eiwit.pdb.

                                                          A not very exiting but mandatory step is:  
                                                            

                                                          |  |  |  |
                                                          | --- | --- | --- |
                                                          |  | grompp -f grompp.mdp -c conf.gro -p topol.top  \| |

                                                            
                                                          This will generate a run input file
                                                          (topol.tpr) from the
                                                          topol.top and conf.gro you generated with
                                                          pdb2gmx.
                                                          A default grompp.mdp is
                                                          available. You can probably use it 'as is', but you might want or need
                                                          to modify some thing. In any case you are encouraged to
                                                          review the description of the
                                                          numerous options in the .mdp file.

                                                          Now, suppose you want to calculate all cross-rmsd values for all
                                                          structures. Enter:  
                                                            

                                                          |  |  |  |
                                                          | --- | --- | --- |
                                                          |  | g\_rms -f eiwit.xtc -s topol.tpr -m  \| |

                                                            
                                                          -f eiwit.xtc and -s topol.tpr are self-explanatory.
                                                          -m tells g\_rms
                                                          to output an RMSD matrix in
                                                          .xpm format, which can be
                                                          directly viewed with for example xv.

                                                          Of course there are many more analysis tools available. For example
                                                          ngmx a trajectory viewer.
                                                          A list of all tools is available in the online
                                                          manual.

                                                          - **Can I fit two structures which do not have the
                                                            same number/sequence of atoms?**  
                                                              
                                                            Yes, just type:  
                                                              

                                                            |  |  |  |
                                                            | --- | --- | --- |
                                                            |  | g\_confrms -f1 file1.xxx -f2 file2.xxx  \| |

                                                              
                                                            g\_confrms accepts
                                                            any generic structure format which
                                                            for instance can be .pdb, .gro or .tpr.
                                                            The program will ask you to select subgroups of both structures for the
                                                            (non mass weighted) LSQ fit. These subgroups must have the same number of atoms, however the two
                                                            structures do **not** need to have the same number of atoms, i.e. two proteins
                                                            with the same number of residues but not the same type of residues can be
                                                            fitted on c-alpha's. You will be warned when the atomnames in the fit groups
                                                            do not match, but the program will go on.
                                                            Option -o gives a .gro file of the second structure fitted
                                                            one the first.
                                                            Option -op gives a .gro file of the two structures fitted
                                                            on top of each other.

                                                          - **I get tired of having to select the same index group
                                                            over and over again. Is there a better way to do it?**  
                                                              
                                                            Use make\_ndx to create
                                                            an .ndx file with only one
                                                            group in it, this is done by typing 'keep #' in make\_ndx,
                                                            where '#' stands for the one group you want to have.
                                                            Name the file index.ndx (which is the default
                                                            filename for index files) and specify the option -n with your
                                                            favorite GROMACS analysis tool. Now this single group will get selected
                                                            automatically every time an index group is needed.

                                                            - **How do I perform an analysis that GROMACS doesn't have a program
                                                              for?**  
                                                                
                                                              We've created a small well-commented template analysis program for you; look in
                                                              /usr/local/gromacs/share/template (or wherever you have gromacs installed.)
                                                              This program reads a topology and trajectory and shows you how to access
                                                              both coordinates and topology information like atom masses and charges.
                                                              There is also a proper GROMACS Makefile in this directory, so if you
                                                              copy the entire contents of the directory you should be able to type
                                                              "make template" to build the program. It's easy to add more programs or
                                                              change the name by editing the Makefile.  
                                                                
                                                              Now, if you wrote an analysis tool which, in your opinion, adds
                                                              something that is really missing in GROMACS, please post it on the
                                                              developers
                                                              mailing list so that all other GROMACS users can also benefit from it!
                                                                
                                                                
                                                              - **My problem isn't mentioned above, and/or none
                                                                of the solutions seem to work?**   
                                                                  
                                                                 Check the installation
                                                                instructions carefully if your problem is related to the
                                                                configuration, building and installation of GROMACS. Also try "Getting
                                                                Started" where a guided tour of GROMACS is provided. A quick
                                                                glance at the flowchart will
                                                                tell you if you missed any essential steps in setting up a run.
                                                                Checking your .mdp
                                                                file against our sample
                                                                .mdp file and the mdp options
                                                                list might solve a number of potential problems. In general it
                                                                never hurts to read the manual pages of all
                                                                the GROMACS programs you (tried to) use. If all this still leaves you
                                                                with any unanswered questions, please post your question to the gmx-users mailing list!

---

http://www.gromacs.org  
gromacs@gromacs.org
